# Flow Injection/Sequential Injection Analysis Systems: Potential Use as Tools for Rapid Liver Diseases Biomarker Study

**DOI:** 10.1155/2012/281807

**Published:** 2012-03-18

**Authors:** Supaporn Kradtap Hartwell

**Affiliations:** Chemistry Department, Xavier University, 3800 Victory Parkway, Cincinnati, OH 45207, USA

## Abstract

Flow injection/sequential injection analysis (FIA/SIA) systems are suitable for carrying out automatic wet chemical/biochemical reactions with reduced volume and time consumption. Various parts of the system such as pump, valve, and reactor may be built or adapted from available materials. Therefore the systems can be at lower cost as compared to other instrumentation-based analysis systems. Their applications for determination of biomarkers for liver diseases have been demonstrated in various formats of operation but only a few and limited types of biomarkers have been used as model analytes. This paper summarizes these applications for different types of reactions as a guide for using flow-based systems in more biomarker and/or multibiomarker studies.

## 1. Introduction

Liver diseases (hepatic diseases) cover the broad range of liver disorders. The liver has many important functions including breaking down toxic substances in the body and excreting them into urine, producing and secreting bile to aid in food digestion, converting glucose into stored glycogen and *vice versa*, metabolizing ingested medicines to obtain active ingredients, and producing blood-clotting factors, amino acids and cholesterol to transport fat. Damage to the liver will cause disruption of these functions which can cause serious damage to the body. Even though the liver is considered the only organ in the body that has exceptional capability in replacing damaged cells, if too many liver cells are damaged, the liver may fail to perform properly. Symptoms caused from liver failure may not be obvious until many liver cells (up to 75%) have malfunctioned [1]. Detection of liver problems at an early stage would increase the chances of curing them. Liver biopsy is currently the best method for diagnosis of chronic liver diseases [2, 3]. However, biopsy procedure involves the rather invasive method of taking a small piece of liver tissue to be examined under a microscope. The repetition of liver biopsy during diagnosis and treatment is hard on patients. Since liver diseases can also affect almost all other body systems, many substances/chemicals in the body may respond to the malfunctioning of the liver. The use of these substances/chemicals as biomarkers has become an area of high interest in research as scientists look for alternative noninvasive diagnostic approaches [2, 4, 5]. Even though none of the biomarkers alone nor the liver biopsy is perfect, they can be used for indication whether further investigation is required [2].

Studies for the effectiveness of biomarkers involve the collection of numerous samples which may be available at different periods of time. Then the analyses of those samples are usually carried out using the same method for comparison. Most of the possible biomarkers being proposed and under study are biomolecules such as proteins and enzymes [6–9]. Analysis of biomarkers in samples with complicated matrices like body fluids requires highly specific and sensitive techniques. Immunoassay is one of the most widely used techniques for these purposes [10]. It can be used to quantify proteins, enzymes, and other biomolecules owing to the flexibility of the immunoassay format and the available antibodies. Fluorescence- and chemiluminescence-based techniques are also of interest due to their high sensitivity [11, 12]. However, these techniques are time consuming and require a skillful operator. From the many possible biomarkers proposed for liver diseases, only a few are accepted for use and only one which is called fucosylated Alpha-fetoprotein (AFP) is a United States Food and Drug Administration approved biomarker [13, 14]. The study of biomarkers for any disease seems to have the same common problem of unreliable results. This is mainly due to having insufficient numbers of sample subjects and insufficient diverse population as well as the lack of reliable and easy methods to manage various sets of samples and to validate analytical methods [13, 15]. On the other hand, since each research group around the world has access to different small sets of samples, it may be useful for the various groups to conduct their studies using different methods. Even though the results cannot be compared directly, the accumulated studies of those results/conclusions about the same biomarker obtained from different analysis methods should help the medical community in drawing conclusions about the trends and effectiveness of a particular biomarker. The effectiveness of the biomarkers should be revealed with similar significance, no matter which methods were used to quantify them.

Analysis technology has evolved into more rapid and low volume operations. The automatic features of the analysis system that can handle tedious and time-consuming analysis protocols are in demand. This paper is focused on the flow-based analysis techniques called flow injection/sequential injection analysis (FIA/SIA) for use as alternative systems for automatic and rapid quantification of biomarkers for liver diseases. The relatively low cost of these semi to fully automatic systems may help broaden opportunities for medical care/clinical studies in remote areas where medical personnel are limited and also provide additional information/results about the effectiveness of the candidate biomarkers.

## 2. Flow Injection/Sequential Injection Analysis

**Figure 1:**
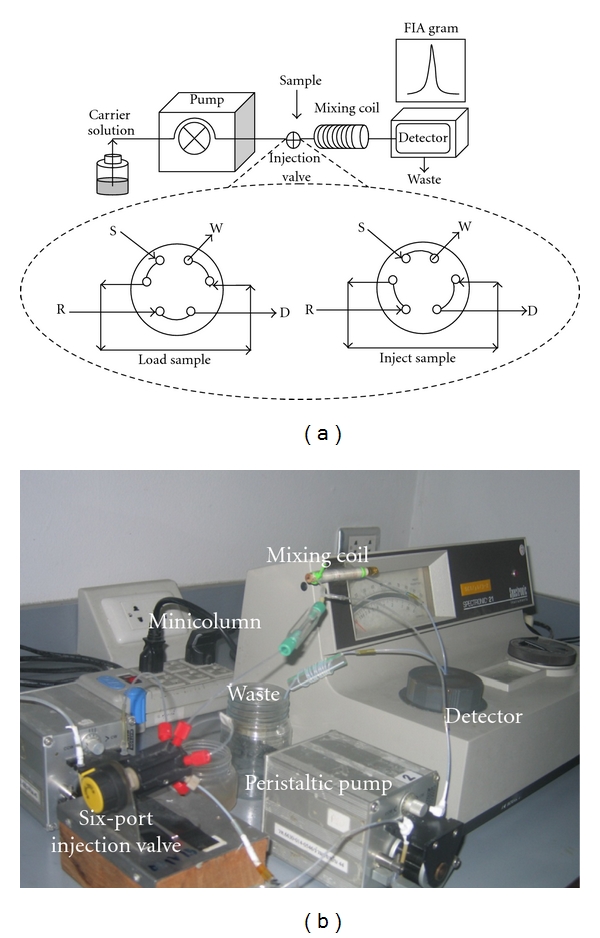
(a) Diagram of a simple flow injection system (S is sample, W is waste, R is reagent, D is detector) and (b) a picture of a simple flow injection system setup showing a peristaltic pump with pump tubing, a six-port injection valve, a minicolumn chemical reactor, a mixing coil, and a detector.

Flow injection analysis technique has been employed to automate a wide variety of chemical/biochemical analyses since its invention in the early 1970s [16]. Owing to its feasibility in coupling with various types of detectors, the applications are numerous. Since parts of the systems can be made in house or replaced as suitable, the cost of the systems is relatively lower than many other commercial instrumentation-based analysis systems. This makes flow injection/sequential injection analysis systems especially suitable for low-budget laboratories. A simple flow injection system, see Figure 1, is composed of a pump for drawing solutions (reagents) into the system via pump tubing. An injection valve is used as an introduction port for sample to merge into the stream of continuously flowing reagent. Detectable product is formed while flowing and simultaneously it enters the detector and then proceeds out to waste. Various types of pump and valve may be used. The most common types are peristaltic pump and a six-port switching valve (similar to one used in HPLC system). Unlike most commercial instrumentation-based analysis systems, the FIA and SIA systems may be built/assembled in house. The beneficial feature of flow injection systems is the flexibility of the formats and designs that can be created based on applications. Various companies offer parts (i.e., pumps, valve, detector, tubing, nuts, and ferrule) for various purposes. These parts can be adapted for use in flow-based systems depending on budget and the flow system design. For example, in the early years of FI development and application in low-budget laboratories, an IV bag hung at a certain height and a cheap aquarium pump were reported as successfully used in place of a peristaltic pump [17]. Lower-cost solenoid valves or plastic 3-way valves and tubing may also be used to construct a hydrodynamic injection system instead of using a more expensive commercial 6-port valve [18].

**Figure 2:**
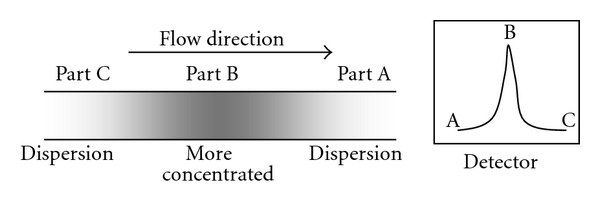
Product zone in the flow line.

Reagent may be used as a carrier, where the sample is injected into the reagent stream. However, if the reagent is high cost and the sample is abundant, reverse FIA [19] may be carried out by using the sample solution as the carrier stream and introducing reagent at the injection point. The product is formed while reagent and sample flow together in the small tubing after merging at the sample injection point. The product zone, see Figure 2, has concentrated product in the middle of the zone, and it is more diluted on both sides of the zone due to dispersion of the solution plug in the carrier stream. Therefore, when the product zone flows through the detector, the beginning of the zone (part A) with low product concentration will enter first, followed by the high product concentration middle zone (part B) and the end of zone with low product concentration (part C). The resultant signal shows as peak signal, called FIA gram, where the highest point resulted from the highest product concentration in the middle of the zone. Detection is done much earlier before steady state; therefore the analysis time is dramatically reduced as compared to conventional batch-wise analyses where detection is normally done after the reaction is completed or has reached steady state. The constant flowrate enables the detection of repetitive analyses to be done at the same point of time. Thus, even though not at steady state, the resultant FIA gram (peak height or peak area) can be precisely related to the quantity of sample.

**Figure 3:**
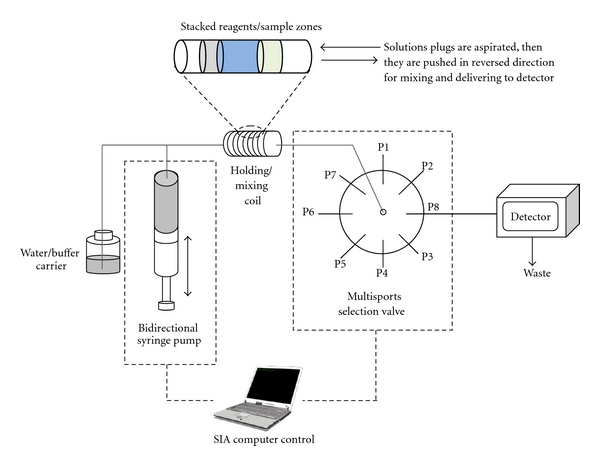
Diagram of a sequential injection analysis system (not to scale). P1–8 are ports on a multiports selection valve for transportation of various reagents.

Later generations of flow injection analysis technique incorporate many pumps, valves, and tubing to accommodate more complicated chemical reactions that need many reagents. The latest generation, called sequential injection analysis [20], see Figure 3, has a downscaled system that consumes even smaller volumes of reagents and samples in a few *μ*L level with the use of a bidirectional syringe pump and multiports selection valve. Reagents and sample can be drawn sequentially and stacked into the mixing coil before mixing while being pushed in reverse direction into the detector. The operational steps from sample introduction, chemical reaction, to detection are fully automated and precisely controlled with computer software. The system can be programmed to stop for a desired period of time; therefore, the study of slow reactions and those that require incubation time such as immunoassay is possible. Accessories such as lab-on-valve (LOV) unit with ports for attaching a fiber optic spectrophotometric detector introduce more areas of applications with real time detection [21–23].

Most research groups have reported that the flow-based systems not only increase sample throughput but also reduce the consumption of sample and reagents. This may be a suitable approach for cases where body fluid/blood samples are limited or need to be divided for various other tests. As compared to most conventional bench top wet chemistry, flow injection requires a lot less sample volume. For example, in titration, sample volume in batch method is in mL whereas in flow-based titration, sample volume injected is in *μ*L [24]. A direct comparison between volumes used can only be made when considering the same analyte and detection methods. Some downscaled batch methods are able to reduce the volume to *μ*L, but in general, FI usually requires relatively less sample volume for a particular analyte or sample being studied. For example, the osmotic fragility test (OFT) of red blood cells normally requires 20 *μ*L of undiluted blood sample in batch spectrometric method whereas only 1 *μ*L of undiluted sample is required in the FI system where it is tested in 100-fold dilution [25]. As compared to standard bioassay technique such as ELISA, the volume required by flow-based systems is also usually lower. For example, the assay of hyaluronan in serum using SI required 10 *μ*L of serum sample, as compared to the conventional routine microplate assay that requires 120 *μ*L of serum sample [26]. For some commercially available ELISA kits such as the cytokeratin 18 (CK18) biomarker kit which requires 50 *μ*L of sample [27], no direct comparison of sample volume usage can be made unless those samples have been tested within the flow-based system, but lower sample volume would be expected in the flow-based system. Most lateral flow chromatography kits may require only a drop of sample, but they normally only yield a simple yes/no answer without any detail of quantity.

## 3. Flow Injection/Sequential Injection Systems as Alternative Tools for Rapid Determination of Biomarkers for Liver Diseases

Table 1 summarizes the works that employed flow injection/sequential injection systems and microfluidics devices for rapid quantitative analysis of some substances that have been reported as candidate biomarkers for liver diseases. Most works emphasize the improved sample throughput. Most works also demonstrated very high precision, reported as percent relative standard deviation (%RSD), as shown in Table 1. Various possible ways of operation and detection using flow-based systems are described.

### 3.1. Flow-Based Analysis System for Simple Reactions with Various Types of Detectors

A flow injection analysis system can be used simply as an automatic system to carry out the mixing of sample and reagent(s). The product formed simultaneously flows into the detector. Depending on the reaction involved, product can be detected by coupling the flow injection system with suitable detectors (e.g., fluorescence spectrometer [28–30], UV-Vis spectrometer [31, 32], Rayleigh light scattering [33–35], or amperometer [36]). Normally, the detection cells for the detectors are modified to be compatible with the continuous flow of solution in the flow systems by having inlet and outlet tubings, and they are commercially available with various volumes and formats [37, 38]. Fluorescence is highly sensitive; therefore, it has been employed as a detector for the analysis systems that involve very low product volume such as in a nanofluidic system [30]. Sakai et al. demonstrated the formation of micelle in the flow line using nonionic surfactant Triton X-100 (amphipathic molecules that arrange themselves in spherical form in aqueous solution with hydrophilic ends outwards and hydrophobic ends inward) [31] and also the successive determination of multianalytes (e.g., human serum albumin and glucose) from the same sample [39]. Even though the samples used in the report were from diabetic subjects, it is clear that the same key idea of multianalytes detection can be adapted for liver diseases cases as well. 

### 3.2. Flow-Based Analysis System for Multisteps Bioassay

A microcolumn packed with specific reagent-coated microbeads can be used as a reactor to accommodate the chemical/biochemical reaction. For example, Gao et al. [40] utilized packed columns with enzyme-coated beads to carry out multisteps enzymatic reactions. Another reported type of reactor [41] is a commercial hollow fiber reactor with cuprammonium rayon membrane for immobilization of enzyme. These works sought detection of bile acids in urine and serum, respectively. Flow injection facilitated the introduction of sample solution and reagent into the reactor and simultaneously transported the colored/luminescent product to the detector. 

Many biomarkers are protein or enzyme which normally can be determined using immunoassay technique. Conventional immunoassay technique is carried out in microplate format where multisteps incubations and washing are done in an array of small plastic wells, each accommodating 100–500 *μ*L volume of solution. The test requires skillful lab personnel to obtain precise and accurate results from handling the microvolume solution and ensuring precise incubation/washing time and volume for each well. The immunoassay process usually takes 3–8 hours depending on detailed steps. The good point of microplate immunoassay format is that many samples can be analyzed in parallel. However, in many circumstances, a small number of samples may need to be analyzed with the demand of quick results. In addition, in many areas of the world, skilled medical personnel are not available to conduct such a complicated test. More automated immunoassay systems where the volume and time are controlled by using a constant flow rate pump to introduce and to draw solutions from the reaction cell have been reported in various formats [42]. 

To change the format from conventional static immunoassay system to dynamic flow formats, the reaction cell has to be changed from being the wall of a microwell plate to other forms of solid surfaces that can be accommodated easily in the flow of solution. Microbeads and capillary are the main types reported. An example for the application in biomarkers research is sequential injection-glass capillary immunoassay [26, 43]. The sequential injection system was used to precisely control the incubation time and small volume of solution in the range of 10–80 *μ*L which is even smaller than some of those used in conventional microwell plate format. A glass capillary was easily connected to the system as part of the tubing that the solution conveniently flows through without any back pressure which may occur when using beads. The wall of the glass capillary was used as the solid surface for immobilization of biomolecules to be employed in subsequent steps of competitive immunoassay. 

Microbeads are used in immunoassay with the capability to increase surface area to improve sensitivity. However, beads that are packed or trapped in the reactor can cause back pressure inside the flow system, so a slow flow rate should be used. Example of sequential injection bead-based immunoassay system was reported for determination of hyaluronan, a possible biomarker for liver diseases [44]. The lab-on-valve (LOV) unit with on-valve fiber optic spectrometer can also be used in conjunction with functionalized beads that are trapped in the LOV unit to obtain direct, real time detection [45]. Although, there is no work reported on its application for study of liver disease biomarkers, the possibility exists for employing LOV bead immunoassay for such a task. Nevertheless, employing beads in the flow system would require more sophisticated control systems than most low-budget laboratories could devise unless pre-existing equipment were to be adapted. 

### 3.3. Flow-Based Analysis System with Preconcentration Capability

The use of microbeads in another aspect, other than using them as a solid surface for immobilization of biomolecules as used in immunoassay, is reported as a preconcentration surface to accumulate analyte of interest before detection. The flow of solution within an easily designed cell for trapping beads and releasing beads when needed is called a flow injection-bead injection system. An example of such a system for alkaline phosphatase in human serum was demonstrated [32]. Even though the work used beads coated with wheat germ lectin for specific binding for bone-alkaline phosphatase, it should be able to combine with total alkaline phosphatase test to estimate for liver alkaline phosphatase. The use of membrane for the same purpose of improvement of sensitivity was also reported. With-state-of-the art development in nanotechnology, nano-pore membrane used with electrokinetic fluid flow as a nanofluidic protein accumulator was claimed to offer a much higher sensitivity in the analysis of human serum albumin than other methods [30]. 

By using a detection method that can measure the differences of surface properties before and after binding to the target analyte, such as surface plasmon resonance (SPR) technique, the amount of bound analyte on the surface could be quantitated directly without the need to add any reagents. Aoki and Toyama [46] demonstrated this system by determination of human serum albumin in urine using gold as an adsorption surface. A flow injection system was employed for continuously feeding the sample containing uric protein onto the gold surface. 

## 4. Remarks

As can be observed from Table 1, most published works that demonstrated flow-based systems for liver diseases biomarkers used the same limited types of target analytes, that is, serum albumin and total protein. There are many substances in body fluids that have been reported as potential biomarkers for liver diseases. Determination of these different biomarkers at the same time may provide better conclusion about the existence of the diseases. Therefore, future studies using flow injection/sequential injection systems should focus on various other possible biomarkers as well as applications for conducting simultaneous detection of multibiomarkers from one shot of sample. Trends in development of analytical devices have also been gearing toward a point of care purpose. The main challenges are to develop the system for solution introduction with controllable flow rates, effective reagent mixing, and detection unit in downscaled format that can be integrated into compact stand-alone devices. 

## 5. Conclusion

Various formats of flow injection/sequential injection analysis and micro-/nanofluidic systems can be set up to study biomarkers. Even though few works have reported on the study of liver disease biomarkers using flow-based systems, works related to determination of protein and enzymes are numerous and should be adaptable for studies of liver disease biomarkers. These flow-based systems are versatile and can be used as an alternative method for rapid screening of biomarkers to aid in disease diagnosis. Their low-volume consumption is particularly suitable for the study of a biomarker, in which samples may be divided for many other tests, either to evaluate different biomarkers or to accompany the initial biomarker test. 

## Figures and Tables

**Table 1:** Summarization of works reported on liver diseases biomarkers using flow-based analysis systems. FI: flow injection; FI-BI: flow injection-bead injection; SI: sequential injection; LOC: lab on chip.

Flow-based system	Detector	Reagent (s)	Biomarker sample	Detection Limit	Working range	Sample throughput	% RSD	Reference no.
FI	Florescence spectrometer	Fluorescein, sodium hypochlorite and surfactant	Albumin in urine	0.03 *μ*g/mL	0.05–24 *μ*g/mL	—	0.8	[28]
FI	Rayleigh light scattering	Amide Black -10B	Albumin in serum	0.11 *μ*g/mL	—	—	<3	[33]
		Dye acid chrome blue K	Total protein in serum	85 ng/mL	2–40 *μ*g/mL	60/h	<2	[34]
		Eriochrome black T		0.8 *μ*g/mL	7–36 *μ*g/mL	90/h	0.76	[35]
FI	Visible spectrometer	Tetrabromophenolph-thalein Et ester triton x-100 (micelle formation reagent)	Albumin in urine	0.05 mg/dL	0.15–12 mg/dL	30/h	1.2	[31]
		Sulfate sulfatase enzyme immobilized on beads packed in reactor	Sulfate bile acid	—	1–75 *μ*M	15/h	<1	[40]
FI	Surface Plasmon resonance spectrometer	Gold surface	Albumin in serum	500 *μ*g/dL	—	90 s/sample	—	[46]
FI	Biolumines-cence spectrometer	coimmobilized luciferase and NADH:FMN oxidoreductase on hollow fiber reactor	3-alpha hydroxyl bile acid in serum	—	1–7.5 *μ*M	>20/h	6–8	[41]
FI-BI	Visible spectrometer	Wheat germ lectin-coated beads and para-nitro phenyl phosphate (PNPP)	Alkaline phosphatase in serum	10 U/L	10–1000 U/L	30 min/sample	5-6	[32]
SI	Visible spectrometer	Hyaluronan standard coated glass capillary, biotinylated HA binding proteins, anti-biotin-HRP and Tetra-methyl benzidine substrate for immunoassay	Albumin in serum	9 ng/mL	Linear 25–500 ng/mL	20 min/sample	3–5.5	[26]
Nanofluidic (LOC)	Fluorescence spectrometer	Fluorescein label	Albumin in serum	0.3 pM	0.3–3 pM	200 s/sample	—	[30]
Microfluidic	Amperometer Glass chip	Substrate conjugated albumin packed in microflow channel	Activity of enzymes (glutamic oxaloace tictransaminase, glutamic pyruvic transaminase, *γ*-glutamyl transpeptidase)	—	Up to 100 −300 U/L	—	—	[36]
